# Virtuous circles: Valuations of plastic recycling in Johannesburg’s inner city

**DOI:** 10.1080/00187259.2025.2499677

**Published:** 2025-06-05

**Authors:** Eileen Moyer, Linda Musariri, Lucy Khofi

**Affiliations:** a Department of Anthropology, University of Amsterdam, Amsterdam, The Netherlands; b School of Public Health, University of the Witwatersrand, Johannesburg, South Africa

**Keywords:** Circular economy, social plastic, social washing, hunger

## Abstract

Imaginings of the circular economy promise to minimize waste and maximize resource efficiency by ensuring that materials are reused, recycled or repurposed in a closed-loop system resulting, ideally, in ‘zero waste’. Drawing on ethnographic research, in this article we analyze the valuation of plastic and food waste within Johannesburg’s inner city, and the moral and material significance attributed to them by various stakeholders, including plastic recyclers, non-profit organizations, property developers and municipal workers. We question the practice of requiring individuals living in extreme precarity to earn food through plastic waste collection, the implications of charging for donated surplus food, the fate of unrecyclable plastic, urban gentrification, and gendered and migrant experiences. Focusing on a community-based program where individuals and families exchange plastic credits for food, we examine political, economic, and moral complexities while critically evaluating the narratives of zero waste and circular economy. We introduce the concept of “social plastic” to highlight how plastic and food recycling are integrated into political and moral agendas in Johannesburg, shaping the greenwashing and social washing practices of different actors.

## IMPLICATIONS

Plastic-for-food programs leave vulnerable communities in precarious conditions, reliant on goodwill rather than sustainable solutions. Circular economy initiatives, while promoting sustainability, often reinforce gendered and socioeconomic inequalities by ignoring structural causes of waste and poverty. Plastics’ non-recyclability and toxic traces create environmental risks, limit scalability, and obscure upstream issues like reliance on oil and gas feedstocks.

## SOCIAL MEDIA STATEMENT

New research questions the moral economy of asking hungry people to trade plastic for food in Johannesburg. What happens when social washing meets greenwashing? #CircularEconomy #ZeroWaste #FoodWaste #PlasticRecycling #Anthropology

**Figure 1.**
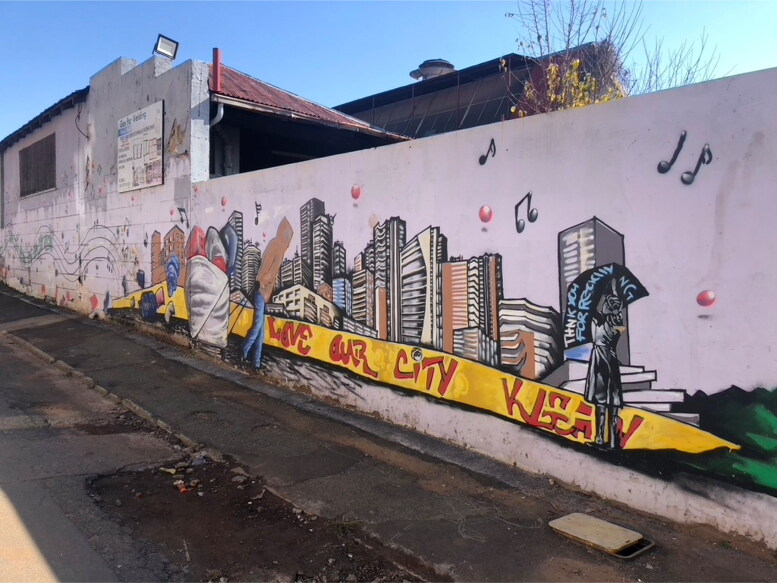
Love Our City Klean mural with a waste gatherer or *bagarezi* in front of the Johannesburg skyline. Photo by Eileen Moyer, june 2022.

Passing by Victoria Yards, an upscale walled collection of shops, restaurants, artist studios, urban gardens, and non-profits in downtown Johannesburg, you might find a line of people queuing up outside a side entrance gate. The way the people are dressed invites you to surmise they are not the usual patrons of Victoria Yards. You may guess they are from the surrounding neighborhood, which in contrast to Victoria Yards, is notably rundown. Mostly women, often with young children in tow, many carry large bags of recycled plastic goods, the size of their bundles belying the lightness of their loads. You might also note the mural on the wall behind the queue (Figure 1). Emblazoned in red on a yellow ribbon that runs across the bottom, passersby and those in the queue are entreated to “Love Our City Klean.” The mural depicts the iconic skyline of Johannesburg with a similarly iconic portrayal of a plastic collector, also known as *bagarezi*, trudging forward under the weight of a cart which residents of the city will know is filled with plastic and cardboard.

Love Our City Klean, also known as LOCK, is a non-profit organization (NPO) that works in partnership with The People’s Pantry (TPP) to facilitate a program in which neighborhood residents can trade plastic for credits to be used for purchasing donated products, ranging from food to sanitary napkins. Interested outsiders who tour Victoria Yards and the surrounding neighborhood are informed that the program was encouraged in part by a Victoria Yards’ visionary committed to brokering “the first zero-waste retail space in Africa.” The tours are offered by the Makers Valley Partnership (MVP), a community-focused and community-facing collective of artists and makers that works to ensure that the virtuous social and environmental aims and claims of the Victoria Yards business developers are both met and promoted.

**Figure 2.**
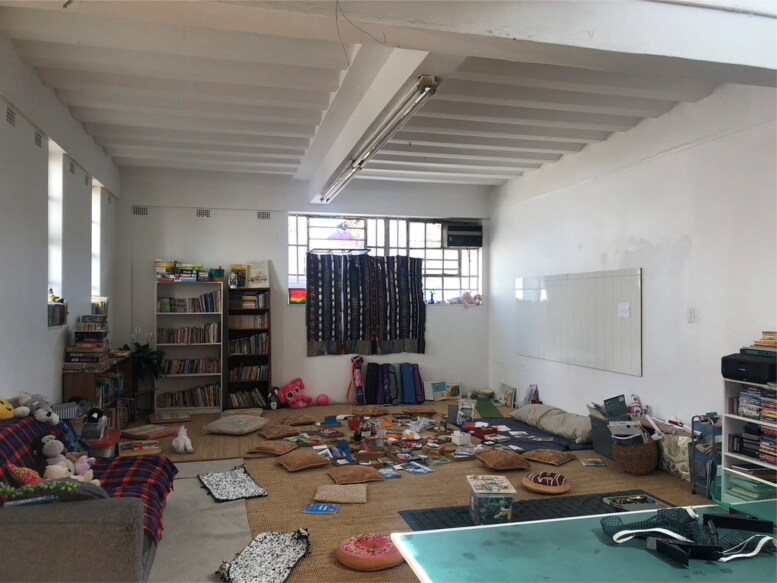
Children’s weekend in daycare located in Victoria Yards and adjacent to the People’s Pantry. Neighborhood children are taught to make crafts from plastic waste. The white bathmats in the lower left are woven from plastic bags. Photo by Eileen Moyer, june 2022.

While the zero-waste goal is admirable, MVP guides will inform those who inquire further that there had previously been a serious problem with unsightly waste piling up outside the walls of Victoria Yards, which was off putting to potential clients and investors alike, nearly all of whom stayed in more upscale parts of the city where waste collection was better regulated. The waste attracted rats and clogged drains when it rained, so something had to be done. The LOCK-TPP-Makers Valley initiative was widely portrayed as ‘win-win’. Hungry people could access food and other essentials on TPP shopping days, and the streets around Victoria Yards would be kept waste free by residents motivated to collect their plastic. The initiative also included an afterschool program for neighborhood children, some of whom could not attend regular schools or benefit from government programs because they were from undocumented migrant families. The school took seriously the mandate to teach children about waste, recycling and other environmental issues, and worked with the children to produce arts and crafts from the plastic waste they collected (Figure 2). It was common to hear employees and volunteers of the different organizations say that the waste-to-food project helped to promote dignity among the poor, giving them an opportunity to trade for food rather than beg.

As ethnographers of plastic and food waste, we admired the vision and enthusiasm of those to whom we initially spoke about the waste-for-food program, but our previous research led us to question what might be glossed over in the ‘just so’ tales we were hearing (Musariri and Moyer 2022). Researchers have shown that most plastics cannot be recycled and that, at least in Johannesburg, it is primarily PET bottles, cardboard and electronic waste that retain value in the recycling industry (Chalfin 2023; Gabrys 2013; Hawkins 2013; Pathak et al. 2024). We presumed that the piles of waste that had previously occupied the sidewalks outside of Victoria Yards contained few PET bottles, as *bagarezi* would have collected them along with other valuable recyclable materials. We had been told that the ability of LOCK to sell the collected recyclable materials to nearby collection depots made the waste-for-food program sustainable, but there were no limits put on the types of plastics that clients could bring for exchange. We wondered what happened to plastics that were brought in that could not be sold on or otherwise recycled, and if LOCK would be able to generate enough capital to compensate its founder-managers or to fund the waste-for-food program.

We were also curious about the purported dignity that was being conferred on those invited to participate in this program. Why were we primarily seeing women and children in the queues? Were people able to adequately address the nutritional needs of themselves and their families through the goods that were on offer? How did they feel about eating food that had been considered waste by others? Finally, we wondered about competition with *bagarezi* who already claimed the territory around Victoria Yards. Known to battle among themselves for access to territories, surely they would not welcome competition.

With this set of questions in mind, we undertook an ethnographic study of the plastic waste for food program, interviewing key people involved in setting up and running the program; assisting and participating during various events where members of the public encountered the program, and in the neighborhood more broadly; and holding informal conversations and interviews with intended beneficiaries. Our objective was to understand the moral economies enfolded into practices related to the enactment of a circular economy in a context of relative poverty and precarity. Most current critiques of circular economies question the promise of zero-waste from an environmental perspective. While we seek to contribute to those debates, we also argue that an ethnographic examination of circular economy practices can help us to understand the limits of such efforts from a social and political perspective.

Drawing on arguments about the relationship between green- and social washing by corporations and businesses, we introduce the concept of ‘social plastic’ to further our analyses of the ways that plastic and food recycling are being enfolded into the moral and political economies of different actors in the city (Adelina and Archer 2024). In conceptualizing plastic as *social*, we want to suggest that even as people become increasingly aware and concerned with plastic waste, plastics are also being reconceived as objects to be manipulated to achieve particular political, moral and aesthetic ambitions, ranging from job provisions for unemployed youth and a currency to underwrite food programs and sustainability initiatives, to teaching children about their environment and transforming neglected neighborhoods into spaces of dignity and pride (de Freitas Netto et al. 2020; Pedregal and Lukić 2024). Such expectations are often unrealistic or can only be achieved in ad hoc ways for the benefit of a few. For those involved in recycling, zero-waste initiatives are quickly confronted with these limits, even when intentions are good and the projects are well thought out, as was the case at Victoria Yards.

While plastics serve as products and packaging for other goods, they also facilitate the circulation of goods in capitalist economies and become waste that fuels recycling, as well as social and green campaigns that obscure the upstream origins of plastics. While crucial, situating plastics in the carbon economy is often overlooked by researchers who focus on waste aesthetics or agency among local recyclers. Hanieh argues that the real issue is not plastics, but the gas and oil industry behind them, urging us to consider “what oil becomes when it’s pulled out of the ground” and highlighting petrochemicals and plastic (Hanieh 2024, 12).

Plastics are ‘leaky’ (Nading 2017), structurally and chemically unstable, breaking down and leaking into the environment and our bodies over time. Most plastics are difficult if not impossible to recycle, and the recycling process itself produces forms of waste that are toxic. Max Liborion (2021) has conceptualized plastics as fitting into the category of “forever chemicals,” pointing to the ways that plastics and other forms of non-degradable waste not only pollute land, air, and our bodies today, but will continue to pollute for generations and possibly forever. Plastics, composed of forever chemicals that leak, create a lasting legacy of contamination that persists even after recycling, affecting both human health and the environment.

Food waste is also unstable, but for different reasons. Without refrigeration or preservation, food products quickly degrade and rot. This is one reason that food wholesalers and retailers in South Africa are keen to donate food that is nearing the end of its shelf life to feeding programs in the city. By donating the food, they hope to avoid waste, but they also avoid waste collection fees and landfill charges. Donating food otherwise headed for the dustbin is imagined to be achieving both an environmental and social good, while allowing food industry players to check the corporate responsibility box and economize on waste management fees. But as we show below, manipulating food waste to combat hunger requires impressive logistics and social framing to ensure it achieves the green and social aims for which corporate donors expect to get credit.

In our work, we are inspired by the field of Discard Studies, which calls for the study of *systems* of waste and wasting on different scales: how something might be framed as waste, or as good or harmful waste (Murphy 2017a, 2017b); and how that might change over time or in different places (Furniss 2017; Strasser 1999). While we are interested in the materiality of waste, whether plastic or food waste, in this article we are more concerned with how different forms of waste are valued within a specific socio-spatial milieu. How does plastic waste come to be construed as unsightly in the context of gentrification? How does the recycling of food waste come to be decoupled from the idea of nutrition (Khofi, Manderson, and Moyer 2024)? How is the labor of recycling configured as both a public good or as an avenue to redemptive citizenship or dignity?

If we consider the plastic-for-waste program through a wider lens of urban abandonment and business driven strategies of gentrification, we begin to understand how current systems of power in inner city Johannesburg are interwoven with material practices that value people, places and things differently. A discard studies approach invites us to pay attention to the *power* at work in these relationships and to examine what *norms* are being (re)produced through well-intentioned programs that attempt to combine green, social and business objectives in one. Through attending to specific forms of waste, we are also able to better understand how power works and to consider “*systems* of discard” (Liboiron and Lepawsky 2022, 7).

We also draw on the work of critical sustainability scholars, including Checker whose research on urban sustainability practices in New York City illustrates the “inherently contradictory promise of urban sustainability: that we can stimulate economic growth while mitigating the effects of climate change, without any sacrifice” (Checker 2020, 7). Checker offers the frame of “environmental gentrification” to illustrate the connection between sustainability initiatives and high-end luxury urban development (Checker 2020, 10), which conceals how the greening of some parts of the city can lead to the browning of other parts of the city (Checker 2020, 14-16). Although Staten Island is worlds away from Johannesburg, her work invites us to reflect on how the abandonment of inner city Johannesburg has been linked to the residential and business development of the leafy, green northern suburbs and how this part of the city came to be developed in specific ways to serve the mining industry, i.e., through building low-income workers’ housing and canalizing the river. Like Checker, we observed that local businesses, non-profits and charities in Lorentzville were invested in rehabilitating the neighborhood but often found themselves reproducing the inequalities they set out to address while concealing larger systems of waste.

## Social plastic or social washing

Although plastics are widely recognized as waste and a pollutant, how plastic as a problem is framed determines how it is presented to the public and what information is highlighted or hidden, influencing how it is addressed (Pathak and Nichter 2019). The UN Habitat’s New Urban Agenda program encourages “residents in developing contexts to see plastic as currency.”^1^ The concept of social plastic began in Vancouver over a decade ago, when the Plastic Bank advocated for plastic waste to be repurposed into new products, thereby improving lives and protecting the environment (Plastic Bank n.d.). Over time, the initiative has grown into a movement supported by individuals, businesses, and NGOs aiming to convert plastic waste into valuable resources to address poverty and environmental crises.

Corporations have seized similar initiatives as part of their ‘green’ efforts through corporate social responsibility (CSR) programs, responding to consumer demand for social and environmental accountability. However, companies often mislead consumers by portraying themselves as environmentally responsible through advertising, without substantiation (de Freitas Netto et al. 2020; Montgomery, Lyon, and Barg 2024). Social washing, as defined by the Environmental Social Governance (ESG) report (2024),^2^ involves companies promoting false social responsibility to profit. This can be achieved through publicly donating to charities working on social issues or sustainability initiatives while withholding important information from the public or covering up negative social impacts, including human rights violations, corruption, or poor labor conditions.

In the case of the plastics-for-food program at Victoria Yards, questions arise about the risks of social washing when the conversion of plastic into currency fails to meet expectations. In community-based initiatives such as these, individual experiences and stories tend to be overshadowed by the collective, when the ‘official story’ enters the public discourse. Social plastic represents a well-intentioned effort to address social, ecological, and business concerns, yet its “social” label often distracts from the need for fundamental change, instead promoting “capitalist techno-fetishism” through green growth narratives (Pedregal and Lukić 2024, 21). Similarly, while food banks address hunger in Johannesburg, they also reflect racial and class-based inequalities. When NGOs and officials promote social plastics as a solution to waste and poverty, they obscure structural issues and perpetuate the illusion that capitalist growth is the answer, while environmental problems are merely manageable side effects (Pedregal and Lukić 2024, 18).

### Moral economies

Moral economy, rooted in economic anthropology, serves as a critical framework for understanding the intricate connections between economic systems and social-moral values. E.P. Thompson’s influential work plays a pivotal role in defining this concept by illustrating how moral norms and shared understandings shaped economic practices within pre-industrial communities. The moral economy recognizes the inherent intertwining of economic transactions with broader social and moral contexts, moving beyond traditional market analyses (Thompson 1971). It emphasizes that economic activities are embedded in a complex fabric of ethical considerations, community values, and shared beliefs, challenging conventional economic viewpoints that often neglect the profound impact of social and moral factors (Thompson 1971; Sosna 2024). The lens of moral economy leads us to uncover the subtle threads of reciprocity, fairness, and justice woven into transactions. This approach fosters a nuanced understanding of how individuals and communities navigate economic challenges while upholding moral principles, highlighting the dynamic interplay between economic structures and the values underpinning social cohesion. In the specific context of Lorentzville, recycling practices have emerged as a tangible expression of the moral economy. Residents, in collaboration with initiatives like LOCK, MVP, and TPP, actively engage in recycling plastics to acquire food items. This community-driven effort not only addresses environmental concerns but also aligns with the moral economy’s principles by promoting sustainability and shared responsibility. Furthermore, the collaboration between LOCK, MVP, and TPP is instrumental in bringing the community together. These initiatives work collectively to clean streets, recycle materials, and foster a sense of shared commitment. The act of recycling is framed as a communal endeavor, reinforcing the moral economy’s emphasis on collective values and ethical considerations. This collaborative effort is not only expected to contribute to a cleaner environment but also to strengthen the social fabric of Lorentzville, exemplifying how community-led initiatives can create positive economic, social, and moral outcomes.

## The research setting

Research for this project took place in Lorentzville, a small neighborhood of mainly one-story residential bungalows located along the canalized Jukskei River on the eastern edge Johannesburg’s central business district. The area has been structurally neglected by the state since the end of Apartheid more than three decades ago; most houses are run down, existing green areas are neglected, and crime and drug use are rampant. While there have been efforts to recuperate the neighborhood, including through the real estate investments of Victoria Yards, the hoped-for gentrification has yet to emerge. Once largely the domain of working-class whites, many of whom fled the city center in search of more secure living in the northern suburbs or other countries, Lorentzville is today filled with what are characterized as “hijacked buildings” (e.g. Wilhelm-Solomon 2020). These are office buildings and residences that have been deserted and now are largely occupied by migrants to Johannesburg from rural South Africa and from other countries. Many of these dwellings lack access to or have been disconnected from municipal services, including water, sewerage and electricity. Because most residents are unofficial, they are unable to register or pay for city services when available, and because most residents do not pay taxes or vote, the city has little motivation to provide services. This includes garbage collection, a primary reason the streets in Lorentzville are often piled with waste.

Lorentzville is an intensely diverse neighborhood, with migrants coming from all over Africa, as well as across South Africa. Most are either marginally employed or not employed at all, and many children do not attend school. The precarity and poverty are entrenched and structural. While it was not uncommon during our research in South Africa, both in Lorentzville and elsewhere, to observe people using the phrase “we are hungry” as an idiom of distress to register a range of social and economic complaints (Khofi, Manderson, and Moyer 2024; Nichter 2010), most of those making use of the waste-for-food program at Victoria Yards did not have access to sufficient food or nutrition.

The People’s Pantry (TPP) first introduced its food program at Victoria Yards at the height of the COVID pandemic, working in partnership with the franchised food chain Nando’s, whose corporate headquarters is located across the street from Victoria Yards, to provide take-away meals to hungry area residents. This hugely popular service allowed Nando’s to avoid food waste at a time when its restaurants were closed due to the pandemic, but this led to some pushback from city public health officials who worried about the risks of people crowded together to pick up meals. Over time, and partly in response to city requests, TPP evolved. At the time of our research, TPP was receiving food donations from multiple sources and was primarily distributing cooked meals through eight decentralized community kitchens. Neighborhood women had been hired or volunteered to cook the meals and TPP provided recycled food and cooking gas. The partnership with LOCK and Makers Valley arose primarily to deal with plastic waste in the community, and as an attempt to put the circular economy aims of Victoria Yards into practice.

Love Our City Klean (LOCK) is a recycling hub that actively engages the community in recycling practices, encouraging keeping the city clean. Its young co-founders, Kei and Zwe, brought their creative art backgrounds and environmental passion to drive this initiative. Their main aim was to create jobs, education, and zero-waste to landfill in Johannesburg by 2025, while enhancing environmental awareness. Each week, recyclables are collected from neighborhood participants who earn points for their contributions. These points serve as currency at the community Swap Shop every Friday, enabling residents to access essential items like food and toiletries. The collected recyclables sometimes take on new life in beautification projects, including “Trash to Art” initiatives and various upcycling projects.

Situated within Victoria Yards, the Makers Valley Partnership (MVP) stands as a community innovation hub, providing a platform to showcase residents’ talents and skills. The moniker ‘Makers Valley’ aptly reflects the vibrant presence and activities of diverse forms of creative entrepreneurship. The community is a rich tapestry of artists, cultural practitioners, artisans, urban gardeners, carpenters, shoemakers, metal and woodworkers, clothing designers, and more. Those we interviewed generally rehearsed the official messaging of MVP, which highlights the harnessing of local creative labor to solve the deeply entrenched problems that characterize Lorentzville. MVP actively promotes a community culture, emphasizing creativity, sharing, learning, participation, mutual support, and positive change. A perusal of MVP’s website (mvcollective.co.za) suggests that the organization’s objectives are rooted in the concept and practice of “changemaking,” and that activities within the Valley are driven by the belief that local entrepreneurs and organizations can “catalyze systemic change within the framework of a well-being economy.” In this neoliberal imaginary, the Valley becomes a nexus of innovation, collaboration, and community-driven progress, propelled forward by the many young people living nearby who, despite having achieved a high level of education, have had trouble finding steady employment. For most of those we interviewed, being part of MVP allowed them to network and apply their creativity and entrepreneurial ambitions toward bettering their own neighborhoods.

In South Africa, municipalities are responsible for waste disposal (Godfrey et al. 2016). Currently, primary waste collection is contracted to waste management companies such as Pikitup, Averda, Environserv, and WastePlan, who work in parallel with informal waste reclaimers (De Kock et al. 2020). Tensions around sub-contracting intermittently lead to strikes of Pikitup workers (2024). Pikitup holds the contract for waste collection in Lorenzville, where services have been negatively affected by the strike. Plastic is considered general waste in South Africa, as opposed to toxic, and contributed 4% to the total waste produced in South Africa in 2018 (Nyika et al. 2019). Since the emergence of the waste recycling economy in 2001 (see Godfrey and Oelofse 2017), only 10% of waste generated has been recycled, with 90% sent to landfill for disposal (Godfrey and Oelefse 2017). The plastic recycling industry in Johannesburg as elsewhere in the country is dominated by informal waste collectors. Some have championed this, characterizing it as win-win, tackling unemployment, plastic litter, and the loitering of street-sleepers (Perlman and Charlton 2021, Musariri and Moyer 2022). The impact of plastic recycling to the South African economy and to the urban environment is evident; plastic recycling has generated profits for some, and the streets are generally cleaner. However, the social impact of plastic recycling is less clear. The waste-for-food program has emerged in a context where community-based plastic recycling had been largely outsourced to *bagarezi*, who also operate in Lorentzville and commonly congregate near Victoria Yards, although they are much more likely to sell their recycled goods at a nearby recycling depot where they can get cash rather than food.

## Methods

Research for this article included ethnographic observations, informal discussions and key informant interviews with a range of actors involved with plastic recycling in Johannesburg, conducted by Moyer and Musariri in 2022 and 2023.^3^ Moyer and Khofi conducted ethnographic observations at Victoria Yards, while Khofi undertook a deep dive into interconnected zero-waste practices of Victoria Yards coordinated by MVP, focusing mainly on plastic and food recycling. This included 30 in-depth interviews with 18 women, 10 men, and two gender-nonconforming individuals from a range of migrant backgrounds. Most women interviewed came from single-headed households, reflecting the socioeconomic landscape. Employment varied, with 21 participants unemployed, one employed at Nando’s head office, and eight engaged in street vending or informal selling. Most women actively participated in zero waste initiatives; most men did not.

Interviews were transcribed directly into English, the most common language used in the multi-ethnic neighborhood. Together with hand-written fieldnotes, these were coded thematically by Khofi before being reviewed by Moyer and Musariri for consistency. Analysis was an iterative process among the three authors who discussed the ethnographic materials at length while developing the arguments of this article. As research collaborations in the community are ongoing, preliminary results were also discussed with key participants during writing.

## Findings

### Victoria Yards/MVP

MVP’s approach to circularity includes reusing resources, managing waste responsibly, and fostering a sustainable and circular community. They see circularity as an inherent good, tied to sustainability, waste reduction, and community well-being. Their ethical stance emphasizes minimizing environmental impact, promoting inclusivity, and creating economic opportunities. One could describe this as an ethical mission, highlighting how their circular practices are entwined with moral values of community making and environmental stewardship. However, as we show, despite good intentions and impressive technical expertise, they face serious sustainability challenges:

As a volunteer at MVP, even though we’re dealing with limited resources, we’re still sticking to our values. We believe in keeping things like waste to a minimum and making choices that are good for the environment and our community. It’s not just a project for us; it’s about being fair, creating jobs, and making our community a better place. Even with our challenges, we’re working hard as a community. We might not reach everyone in the whole Valley, but we’re doing our best to make a positive impact right here in our community.

This response was typical of young people involved in promoting the circular economy vision, enthusiastic and hopeful while also fully aware of the “challenges” they faced. MVP recognized that some individuals engaged in recycling primarily to secure meals rather than to keep the streets clean, and that some felt uneasy about associating with waste to access food. Actively participating in the project by attending recycling and waste workshops, engaging with educational graffiti, and contributing to community projects, they argued, allowed community members to imagine themselves as contributing to a greater good, rather than collecting waste for the sole purpose of putting food on the table. These initiatives also fostered a sense of community involvement beyond the immediate goal of securing meals.

The volunteers involved in running the program, largely made up of educated, lower-middle class long-term residents of the Jukskei Valley, believed in the community’s responsibility to care for the city. While they expressed a desire for companies to reduce waste production and use recyclable materials, they also acknowledged that some materials were not recyclable. In response, they channelled their creativity into repurposing non-recyclable items, such as melting plastics to craft artificial flowers for Victoria Yards’ decoration and to craft plastic bottle planters. Although there seemed to be limited markets for these crafts and little awareness of the toxic exposures from melting plastics or shedding microplastics in craftwork, those we interviewed remained largely optimistic that through creativity, residents could overcome the impact of wider systems of waste and waste management. One MVP volunteer said:

Community members must take responsibility for maintaining a clean environment and protecting their city. Instead of solely blaming the municipality for neglect, it’s crucial that we take proactive steps to address the issue. The underlying concept involves encouraging community members to take charge of their surroundings. In doing so, not only do they contribute to a cleaner environment, but they also reap benefits in the form of food items, fostering a sense of collective responsibility and mutual benefit.

### The people’s pantry

On a particularly hot day, we witnessed community members, mainly women, cleaning the streets near Victoria Yards. They extended their efforts to clean along the Jukskei River, focusing mainly on plastic waste. Although they were not being paid, they diligently collected and stored the plastics for delivery to the LOCK depot where they would receive credits to be used at TPP. Although MVP plays a key role in launching and promoting green and zero waste initiatives in and around Victoria Yards, the day-to-day work of the plastic waste-for-food program is carried out by LOCK and TPP, with limited resources.

In 2021, TPP entered into a collaborative agreement with LOCK to establish a swap shop to enable community members to earn food parcels. While several community kitchens provide meals every Tuesday, not everyone is able to access these meals. TPP workers are aware of hunger within the community and seemed most concerned with supporting women who had young children at home. TPP volunteers, many women who participated in recycling to earn points for purchasing at TPP, also received food parcels. Some reported that they engaged in recycling because they were sole providers in their households; others did so out of concern for their community. Most reported that they appreciated the opportunity to learn more about the environment and recyclable materials:

When we initially launched the swap shop, some individuals faced challenges as they were not familiar with recycling. The task of creating trolleys and actively seeking out waste appeared daunting until LOCK organized multiple meetings. These sessions raised awareness about maintaining clean streets and provided education on identifying recyclables and understanding the recycling process.

**Figure 3.**
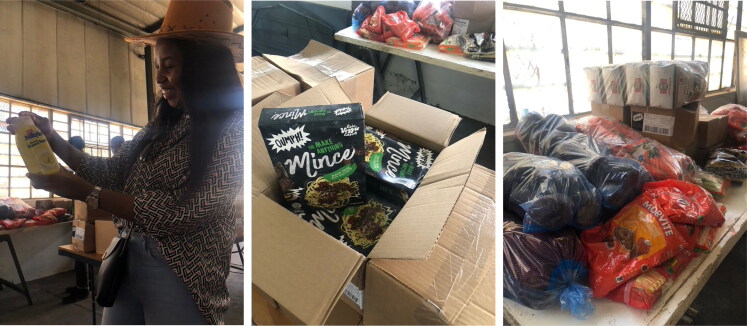
4 and 5: Inventory of goods delivered by Harvest SA to The People’s Pantry, including a sugary syrup to be added to milk, soy mince, and assorted vegetables and grains. Photo by Eileen Moyer, june 2022.

While TPP supports the initiative, it recognizes that there are times when there is not enough food items for distribution. During one Friday observation at the swap shop, a limited supply of vegetables, including cabbage, potatoes, carrots, and one kilogram of maize meal, attracted people as they were arriving from the LOCK depot (Figures 3–5). The available food items were quickly depleted, and a woman carrying a child pleaded with a TPP volunteer, saying that she had nothing to eat at home and had worked (gathering plastics) that day to accumulate more points. The volunteer replied:

You’ve earned more points than anyone today, Mama, indicating your hard work. I apologize, but we’ve run out of food items, as you can see. You can use your points next week on Friday. However, if we receive any donations during the week, I will personally bring the food items to your house.

TPP depends solely on donated food from organizations such as Harvest, Nutripick, and local small-scale urban gardens. When we were conducting fieldwork, Harvest SA delivered food items every Tuesday morning, mostly fruit and vegetables often nearing its sell-by date. The bulk parcels would be divided up by volunteers to be used in the community kitchens, with effort made to provide the basics of a nutritious meal. However, there was often a shortage of key ingredients, including starches, cooking oil, spices, meat, or other sources of protein. Volunteers also collected their personal food parcels on Tuesdays. Anything that remained would be set aside for the Friday swap shop, but often there was little left or so much of a particular item that it risked spoiling before it could be consumed. Organic fruit and vegetables that could not be consumed were collected for compost and eventual use in the community gardens in Victoria Yards. In 2023, TPP also began a food preservation program to conserve surplus fruits and vegetables.

Harvest SA operates on the principle of ‘rescuing’ food destined for the waste bin and municipal landfills, aligning with TPP’s belief that rescuing and preserving food can address hunger in the community. Harvest SA collects foods from supermarkets and agricultural businesses, and delivers these to community outreach programs using refrigerated trucks. However, they having little control over food quality or quantity. While their work is laudable, any given shipment provides material insight into the wider systems that result in food waste in South Africa and how food recycling programs are decoupled from nutritional goals.

One Tuesday, we observed the delivery of 10 cases of fresh vegan mince and six cases of a sugary syrup meant to be mixed with milk for a treat; no milk was provided (Figures 3 and 4). Both the mince and the syrup, like much of the prepared food, came in plastic packaging, most of which would not be recyclable. While vegetarianism is not uncommon in South Africa, we wondered how welcome plant-based protein would be to people who regularly lamented they were hungry for meat. As we struggled to fit the mince and other food into the refrigerators, one volunteer commented that there would be trouble if the electricity went out, a frequent occurrence at the time. Another commented that one could not really trust expiration dates, since so much food in warehouses and shops was subjected to regular electricity outages. TPP is committed to helping people facing critical food shortages, but its work is limited by the wider systems at work in the city.

### Lock

The co-founders of LOCK recognized the need for the waste-for-food program through their involvement with MVP and the proximity of their offices to Victoria Yards. LOCK is an award-winning local recycling initiative motivated to take and promote responsibility to keep the inner city clean in the absence of state services. According to LOCK’s female co-founder:

Residing in the city has taught us not to depend on government or local municipality services. Having experienced communities where waste was neglected, our passion for repurposing discarded materials and creating something new brings us immense joy. Collaborating with local artists adds a creative dimension, showcasing the various possibilities of repurposing waste and raising awareness about recycling within the community.

LOCK founders and volunteers see the waste-for-food program as a community project. The male co-founder expressed, “Sadly, most of the willing participants are women. Some men cite concerns about their dignity, expressing reluctance to be seen recycling for food items. They prefer recycling and taking materials to a depot that provides monetary compensation rather than points.” Although most interviewed expressed appreciation for the project, there have been instances of vandalism against the community dustbins created by the community and LOCK.

### Community members

Many community members we interviewed engaged in zero-waste initiatives, with 22 out of 30 individuals, the majority women, participating. The remaining eight, mostly men, did not partake in any waste or recycling initiatives in the community. Most referred to collecting reusable materials as “recycling,” underscoring how waste pollution negatively affected their streets and the community at large. Some participants drew connections between waste issues and climate change, expressing concern about the environment’s ongoing struggle against human activities: “The more we neglect the environment – the more it will turn against us; now the environment is a beast.”

**Figure 6.**
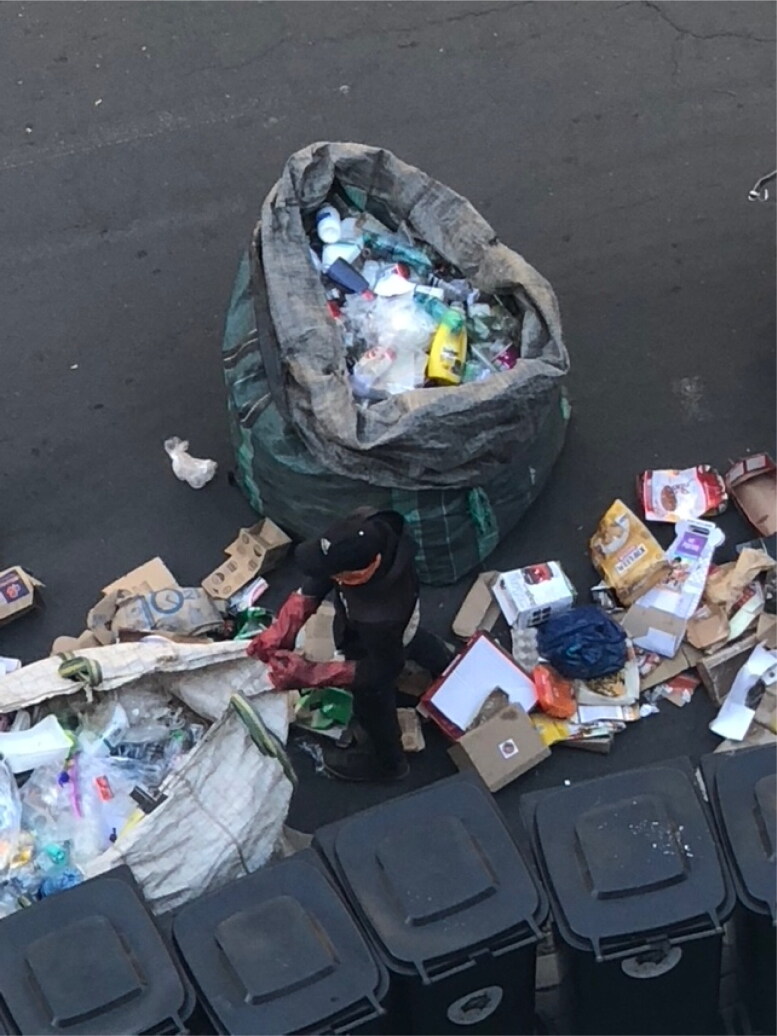
A waste collector or *bagarezi* sorting out plastic and cardboard from curbside bins. Photo by Eileen Moyer, January 2023.

**Figure 7.**
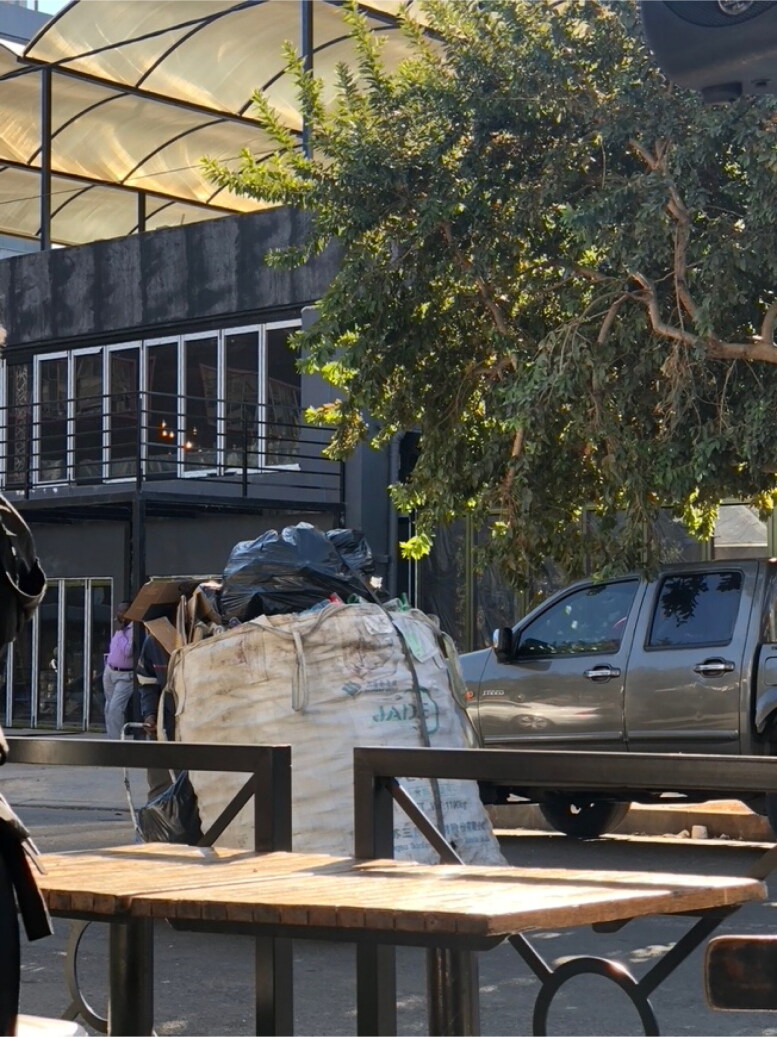
*Bagarezi* carting collected materials through downtown Johannesburg on the way to a recycling depot. Photo by Eileen Moyer, January 2023.

A few participants viewed recycling negatively, considering it dirty and associated with individuals of lower socioeconomic status or those experiencing homelessness (Figure 6; Figure 7). They expressed a lack of interest in recycling initiatives and perceived these as time-consuming and unproductive:

In this community, those dealing with waste are seen as struggling to make ends meet. It’s like a symbol of poverty. People engaging in waste collection often face judgment and stigma, making it difficult for anyone to openly participate in such activities without feeling a sense of shame.

**Figure 8.**
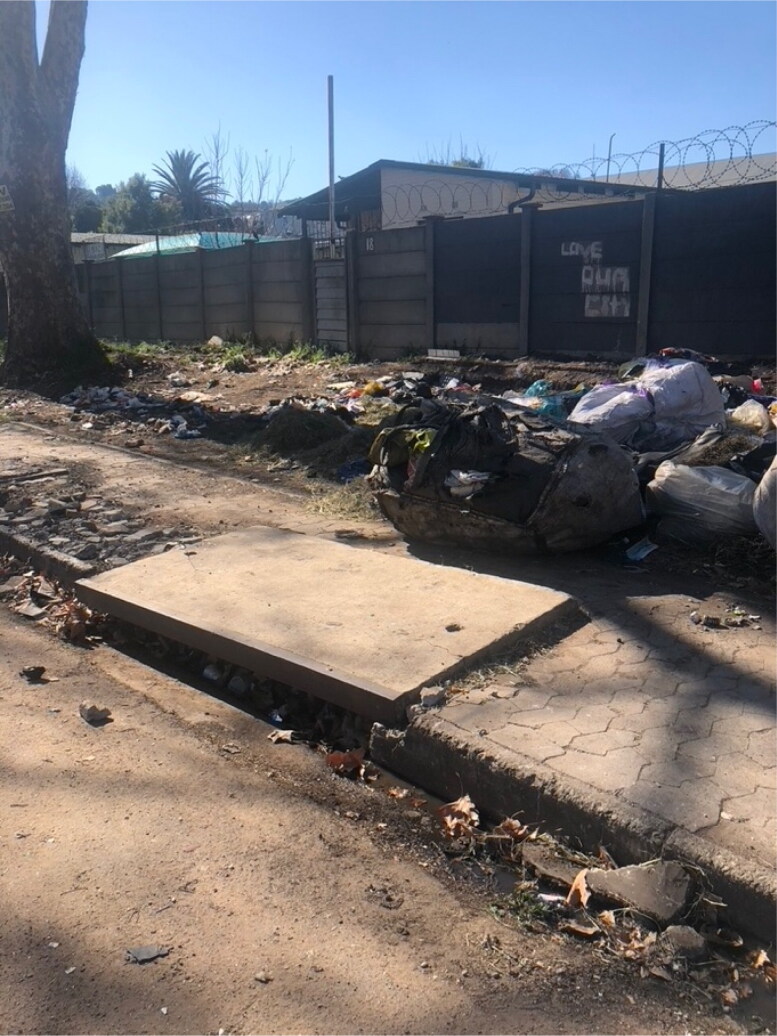
One city block from Victoria Yards, abandoned (non-valuable) plastic waste is discarded on side of road in front of “Love our city” graffiti that may be part of the Love our city klean initiative. Rubbish is clogging the sewerage drain here, increasing risk of urban flooding. Photo by Eileen Moyer, June 2022.

As you walk through Lorentzville, you encounter Love Our City Klean signs and artwork near waste dumping spots (Figure 8). These artistic interventions serve as reminders for community members to prioritize a clean city. Despite these efforts, some do not accept that it is their responsibility to maintain a clean environment, relying instead on Pikitup, the company contracted by the municipality to handle rubbish collection. Residents tend to blame “the city” or “Pikitup” when asked about waste in the streets. The city explains these failures as a consequence of population growth in Lorentzville, which it claims results in quantities of rubbish that surpass the capacity of the designated municipal waste collection plans:

It is so sad that people do not care about this city! This area does not mean anything to them. I don’t know. It feels like women are the ones who care about this place, we do whatever it takes to protect our city. This includes keeping our city clean… I love the initiative by LOCK. They keep on encouraging us to clean our city. When they invite local artists to draw nice graphics in the streets where we are dumping the waste; it means so much for us to have such a support system to love our city.

While some community members recognized the potential economic value of recycling as they earned points to purchase essential food items, others chose to take their collected materials directly to the depot to earn cash instead. When asked if they would engage in recycling without compensation, most responded negatively.

In Lorentzville and elsewhere in Johannesburg, men primarily collect and deposit waste at depots. Individuals engaged in waste collection often appear unkempt, wearing torn clothes. Some described recycling and recyclers as dirty. Such perceptions contributed to the stigma around waste collection. People living on the streets around Victoria Yards reported that recycling provided an income for them. Some expressed that recycling also offered a sense of purpose.

## Discussion

In this study, we set out to examine an attempt to enact a circular economy in a neighborhood characterized by entrenched poverty and state abandonment. We documented strong enthusiasm for the program. In the wake of other nearby gentrification efforts (i.e., Maboneng), Victoria Yards emerged as an experiment in urban renewal that strives to include the surrounding community and aims to be carbon neutral. Primarily driven by private capital, MVP is keen to work with businesses to create jobs and to stimulate youth in the area to embrace entrepreneurialism. The zero waste initiatives, including the waste-for-food program, are morally driven by the idea of communities taking responsibility for their own neighborhood and educating community members about sustainability, good nutrition, and waste management, among other things.

When pressed to discuss challenges to the zero-waste ambition or the waste-to-food program, those we interviewed tended to downplay the challenges they encountered or to frame them as logistical or funding issues. We rarely heard people discuss the economic drivers of the recycling industry, in which context only specific waste materials and plastics have value. At the time of our research, LOCK was struggling to cover the rent costs for the warehouse where it stored the plastics it could not sell; we only learned about this by chance because everyone we formally interviewed only spoke positively about the program. As noted above, while some of these plastics were used to create crafts to be sold at the monthly Victoria Yards’ market, these were not particularly popular items, and the quantities of unrecyclable plastic far exceeded the demand for upcycled plastics. There was no mention of the potential toxicity of these upcycled items in our interviews. When we spoke to people, most glossed over the amounts of valueless plastic accumulating, and made no mention that the market value of recyclable plastics had dropped in recent years.

What happens to the bread bags and polystyrene yogurt tubs that have no value on the market? Do these end up as landfill, left to blow about on the streets, burned in people’s backyards, or pile up in a warehouse somewhere?

MVP is in an ideal position to document the difficulties of enacting a circular economy. Its members are widely sought out as speakers for sustainability initiatives and climate change. High level delegations from wealthy countries and INGOs routinely incorporate site visits to the Instagram-friendly Victoria Yards to pose in front of garden beds of near perfect kale, too expensive for neighborhood residents. Although the stories they share with the world tend to downplay the challenges we have documented, we would argue the importance of understanding these challenges if sustainability and carbon neutrality are to be achieved.

Those we interviewed from TPP were more up front about the challenges they faced in terms of logistical issues, food quality and quantity, waste, and the failure of the municipality to provide basic services for people who were often classified as migrants or ‘illegal’. The food donations they received were often inadequate or inappropriate. Their goal was to provide nutritious meals to those in need, and they were often confronted with a shortage of basic ingredients such as cooking oil and carbohydrates like rice or maize meal. They did not know what was coming until the truck was unloaded. While they did have refrigeration to store perishables, they sometimes ran out of space. Given ongoing power outages, there was also the risk that food might go bad. Power outages led to suspicions that some food was donated because retailers could not keep foods adequately refrigerated. They worried that the food they distributed could make people sick.

The challenge faced by TPP can be directly linked the corporate social responsibility (CSR) model that benefactors such as Nutripick and Harvest have taken in their approach to contribute to green initiatives. The CSR model works as a charity and takes away obligation and responsibility from the donors. Researchers of plastic recycling have criticized the private sector’s use of a charity model, which presents informal collectors as unknowledgeable charity cases rather than eligible wage laborers (Iqani 2020).

A challenge noted by representatives from all three organizations and community residents was that many men refused to participate in the program, which they described as beneath their dignity or ‘unmanly’, even though research with *bagarezi* routinely described the work as most suitable for men (even if some women did participate) because those who did it were required to walk long distances and at times work overnight (Iqani 2020; Musariri and Moyer 2022; Samson 2025). Tying plastic recycling to a food program appears to have feminized the project in the eyes of men living in the area, likely because securing food for the family is thought of as women’s responsibility. The men we talked to who practiced recycling preferred to take the plastics directly to the depot to collect cash or else sent their children to TPP to collect points and food parcels. We also heard stories of some people selling their food parcels for cash or trading food for drugs, although we did not directly observe this. A program established to instill dignity among those in need was experienced as undermining the dignity of men in the community.

The waste-for-food program and the circular economy ambitions of Victoria Yards has been widely touted as an example of sustainability in practice, and those involved are considered champions for combatting climate change. Although some who collect plastics or cook meals expressed an interest in improving their immediate environment, they were primarily involved in the program to gain access to meals. Similarly, previous work with *bagerezi* demonstrated that even though they were presented as climate change champions by corporates and city sponsors, none we interviewed cared about climate change and few made connections between their work and broader global discourses. The *bagarezi* we interviewed reported that they engaged in the work primarily to secure a meal. Sosna’s (2024) research on the moral economies of waste management provides a comparative case illustrating how contemporary engagement with waste provides a lens for understanding situational ethics and struggles for dignity in an unequal world.

In Lorentzville, socially focused environmentalism seems geared toward making the neighborhood a cleaner and safter space, which is hugely beneficial for Victoria Yards’ investors and businesses. Although the program alleviates extreme hunger for a select number of residents, it also conceals the market forces driving both hunger and waste. There is a risk that such programs might exacerbate existing inequalities and undermine genuine efforts to promote sustainability and social justice. Resorting to social plastic as a solution to plastic waste focuses attention and effort on downstream solutions without questioning the broader structural and capitalist factors that drive both pollution and the plastic-for-food program – factors which Discard Studies insists that we must consider. Downstream solutions have been criticized as neo-liberal responses that background the economic drivers of pollution, making it impossible to adequately address the broader challenges associated with climate change (Gibbens 2019). In light of these limitations, social plastic is at risk of fueling social washing, diverting attention from the root cause of pollution and the “overconsumption of resources” (Stafford and Jones 2019,187).

## Conclusion

In this article, we examined a project aimed at implementing a circular economy within a context of poverty and precarity. Inspired by Discard Studies, we also considered what broader structures, systems and mechanisms are concealed or reinforced by interventions that claimed to address environmental and social issues. Our findings demonstrate that initiatives framed as being for the collective good – street cleanliness, civic pride, environmentalism, zero-waste and recycling – often overlooked individual experiences and motivations when describing the value of the project. Street clean-ups, recycling projects and community kitchens emerge as moral spaces in which community members perform commitment to the collectivity and, in connection, their deservedness as recipients of food-based charity. Those responsible for organizing and operating such initiatives are largely motivated by a perceived need to address serious service gaps resulting from a largely absent state. We see this moral economy as a street level manifestation of a larger moral economic framework unfolding through a range of green and social initiatives that work to conceal the upstream causes of waste, pollution and poverty.

We have introduced the concept of “social plastic” to describe plastic recycling initiatives, such as the plastic-for-food program we documented, framed as solutions to social issues like hunger, unemployment, undocumented migration, or municipal failures. Social plastic can be linked to social washing, where corporate, state, and community actors obscure deeper issues. A key challenge is the materiality of plastic—most plastic is non-recyclable due to market and technological limitations and even recycled plastic leaves toxic traces. While projects like Victoria Yards creatively repurpose plastic, their solutions are short-term and not scalable. Corporate support for plastic recycling often relies on a charity model, sidestepping accountability and creating precarious conditions for beneficiaries, as seen in food shortages in our study. This model fails to meet participants’ nutritional needs, leaving them dependent on the goodwill of others. Despite well-intentioned goals, the project perpetuates stigma and shame among some participants.

We align with de Sousa Santos’ critique that green initiatives reframe issues to appear solvable, while preserving capitalist modes of production. Our aim is not to critique local actors in Lorentzville, Johannesburg, but to reflect on how zero-waste initiatives are structured to prevent critiques of the broader structural issues. Despite the positive short-term impacts of these initiatives, we stress the importance of addressing upstream practices that reproduce social and ecological problems. Those we engaged with were optimistic about their work but also recognized its limits and the need to challenge the production, consumption, and disposal of plastic at larger scales. We question why plastic waste continues to accumulate in certain neighborhoods, and why individuals present to food banks and opt for food in exchange for plastic instead of work. Shifting attention upstream reminds us that 90% of plastic production relies on oil and gas feedstocks, which accounted for 6% of global oil production in 2019 (Singh and Walker 2024).

Initiatives aimed at fostering a circular economy must be analyzed within a broader social context that addresses the systems and structures of waste. In our research the diversity of individual experiences, the moral implications of charity-based models, and circularity efforts all perpetuate gendered and other inequalities. Acknowledging these complexities is essential for creating sustainable solutions that genuinely benefit vulnerable communities.

## Notes

1
https://www.urbanagendaplatform.org/best-practice/social-plastic.

2
https://www.esgthereport.com/what-is-social-washing/.

3 Moyer and Musariri conducted targeted observations and interviews on waste recycling at Victoria Yards and elsewhere in Johannesburg during five two-week field visits in this period, while Khofi conducted research at Victoria Yards over a year while engaged in a larger ethnographic study related to her PhD.
